# Peptidoglycan Hydrolases RipA and Ami1 Are Critical for Replication and Persistence of Mycobacterium tuberculosis in the Host

**DOI:** 10.1128/mBio.03315-19

**Published:** 2020-03-03

**Authors:** Claire Healy, Alexandre Gouzy, Sabine Ehrt

**Affiliations:** a Department of Microbiology and Immunology, Weill Cornell Medical College, New York, New York, USA; University of Massachusetts Amherst

**Keywords:** cell division, pathogenesis, tuberculosis

## Abstract

Synthesis and cleavage of the cell wall polymer peptidoglycan (PG) are carefully orchestrated processes and are essential for the growth and survival of bacteria. Yet, the function and importance of many enzymes that act on PG in Mycobacterium tuberculosis remain to be elucidated. We demonstrate that the activity of the *N*-acetylmuramyl-l-alanine amidase Ami1 is dispensable for cell division in M. tuberculosis *in vitro* yet contributes to the bacterium’s ability to persist during chronic infection in mice. Furthermore, the d,l-endopeptidase RipA, a predicted essential enzyme, is dispensable for the viability of M. tuberculosis but required for efficient cell division *in vitro* and *in vivo.* Depletion of RipA sensitizes M. tuberculosis to rifampin and to cell envelope-targeting antibiotics. Ami1 helps sustain residual cell division in cells lacking RipA, but the partial redundancy provided by Ami1 is not sufficient during infection, as depletion of RipA prevents M. tuberculosis from replicating in macrophages and leads to dramatic killing of the bacteria in mice. Notably, RipA is essential for persistence of M. tuberculosis in mice, suggesting that cell division is required during chronic mouse infection. Despite the multiplicity of enzymes acting on PG with redundant functions, we have identified two PG hydrolases that are important for M. tuberculosis to replicate and persist in the host.

## Abstract (precis)

Tuberculosis (TB) is a major global heath burden, with 1.6 million people succumbing to the disease every year. The search for new drugs to improve the current chemotherapeutic regimen is crucial to reducing this global health burden. The cell wall polymer peptidoglycan (PG) has emerged as a very successful drug target in bacterial pathogens, as many currently used antibiotics target the synthesis of this macromolecule. However, the multitude of genes encoding PG-synthesizing and PG-modifying enzymes with apparent redundant functions has hindered the identification of novel drug targets in PG synthesis in Mycobacterium tuberculosis. Here, we demonstrate that two PG-cleaving enzymes are important for virulence of M. tuberculosis. In particular, the d,l-endopeptidase RipA represents a potentially attractive drug target, as its depletion results in the clearance of M. tuberculosis from the host and renders the bacteria hypersusceptible to rifampin, a frontline TB drug, and to several cell wall-targeting antibiotics.

## INTRODUCTION

Tuberculosis (TB) is the current leading cause of mortality in the world due to a single infectious agent, causing 1.6 million deaths annually (1). Mycobacterium tuberculosis, the causative agent of tuberculosis, owes a lot of its success as a pathogen to its complex cell envelope. The thick waxy coat provides an impermeable barrier that is composed of lipids, including mycolic acids, a defining feature of mycobacteria that is capable of modulating the host immune response (2, 3). Many components of the cell envelope provide attractive targets for drug development. In fact, the current chemotherapy regimen for treating TB includes two drugs, isoniazid and ethambutol, that inhibit the synthesis of cell envelope mycolic acids. Improving our understanding of how the components of the cell envelope are generated, maintained, and modified may provide novel drug targets for antimycobacterial drug development.

**FIG 1:**
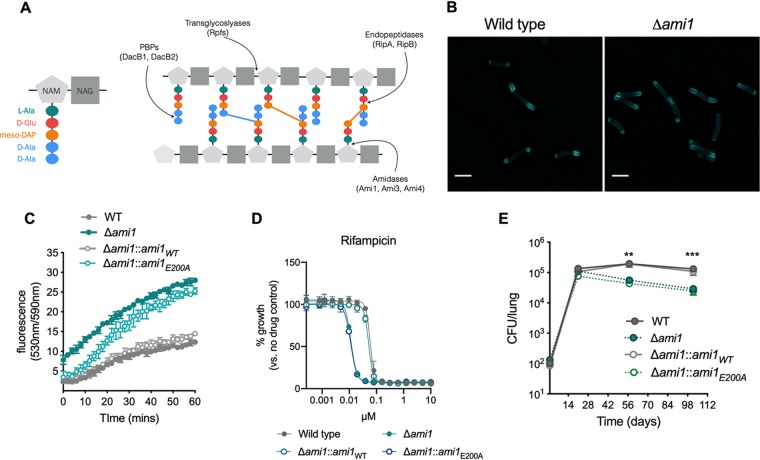
The peptidoglycan amidase Ami1 is dispensable for M. tuberculosis cell division *in vitro* but is important for persistence *in vivo*. (A) Schematic diagram of peptidoglycan (PG). *N*-Acetyl muramic acid (NAM) and *N*-acetylglucosamine (NAG) residues make up the glycan backbone of PG. The pentapeptide stem constitutes of l-alanine, d-glutamate, meso-diaminopimelic acid (mDAP), and two d-alanine residues (left panel). Different classes of PG-hydrolyzing enzymes cleave the PG macromolecule at different sites (right panel). The colored bars between peptide stems represent cross-links (blue, 4-3; orange, 3-3). (B) Micrographs of M. tuberculosis cells labeled with the fluorescent d-alanine analogue HADA for 20 h. Bar, 2 μm. (C) Ethidium bromide (EtBr) uptake assay. Bacteria were incubated with EtBr, and the fluorescence (indicating uptake of EtBr and binding to DNA) was monitored over the course of 60 min. (D) MIC profiles of Δ*ami1*, Δ*ami1*::*ami1*_WT_, and Δ*ami1*::*ami1*_E200A_ strains relative to that of the wild type for the frontline TB drug rifampin. (E) Bacterial burden in the lungs of CB57BL/6 mice infected by aerosol inhalation with the following strains: wild-type H37Rv (WT), Δ*ami1*, Δ*ami1*::*ami1*_WT_, and Δ*ami1*::*ami1*_E200A_ variant. Significance was determined by one-way analysis of variance (ANOVA) and adjusted for multiple comparisons. **, adjusted *P* (adj-*P*) < 0.005; ***, adj-*P* < 0.0005. All data are representative of two independent experiments. Data in panel E are from 2 independent experiments, each with 4 mice per group and time point.

Peptidoglycan (PG), the rigid polysaccharide layer that lies between the outer “mycomembrane” and the plasma membrane, is composed of multiple strands of a glycan backbone comprising repeating disaccharide units of *N-*acetylglucosamine (NAG) and *N*-acetyl-muramic acid (NAM) (Fig. 1A). Peptide stems project from the backbone, anchored at the NAM disaccharide, and are cross-linked to one another, providing the strength and rigor of this mesh surrounding the cell. PG is unusual in mycobacteria as it contains predominantly 3-3 cross-links between the third residues of the peptide stems (Fig. 1A) (4, 5). These 3-3 cross-links are formed by l,d-transpeptidases (LDT), PG-maturing enzymes, and are important for the survival of the bacterium in the chronic stage of infection (2, 3, 6, 7). Although PG is rigid, it is not a static structure; it must be expanded, broken apart, and modified during the cell cycle to allow the cell to grow and divide (8). Indeed, during cell elongation, the existing PG macromolecule must be cleaved for the incorporation of new PG material near the poles. Also, during the final steps of cell division when the septum is formed, PG needs to be cleaved to allow the physical separation of two daughter cells. Thus, the synthesis and cleavage of PG during cell growth and division is a carefully coordinated process that allows PG to remain intact and retain the cellular structure. Several enzymes (PG hydrolases) are capable of cleaving PG and do so at different positions on the macromolecule (Fig. 1A). Lytic transglycosylases, such as the resuscitation-promoting factors (Rpfs) are believed to cleave between the NAG and NAM disaccharides of the glycan backbone (9–11). d,l-Endopeptidases such as RipA and RipB cleave within the peptide stem (12), while NAM l-alanine amidases cleave the peptide stem from the NAM residue on the glycan backbone (Fig. 1A).

The M. tuberculosis genome encodes four predicted NAM l-alanine amidases, Ami1 (Rv3717), Ami2/CwlM (Rv3915), Ami3 (Rv3811), and Ami4 (Rv3594) (13). Recent studies have discovered that Ami2/CwlM, which is essential in M. tuberculosis and in Mycobacterium smegmatis (a nonpathogenic and fast-growing mycobacterium), lacks amidase activity but instead regulates cell growth within the cytoplasm by interacting with several PG biosynthesis enzymes, depending on its phosphorylation status (14, 15). While the activities of Ami3 and Ami4 remain unknown, the crystal structure of Ami1 has been solved by two independent groups, and Ami1 PG hydrolase activity has been demonstrated *in vitro* (16, 17). Interestingly, M. smegmatis cells lacking the Ami1 homologue (MSMEG_6281) form multiseptated chains with a high frequency of lateral branching, indicative of a cell division defect (18). However, the role of Ami1 in cell division in M. tuberculosis has not yet been investigated.

Redundancy is often observed for PG amidases in other bacteria, such as Escherichia coli, where deletion of several amidases is required before substantial defects in cell division are observed (19). While the genome of M. tuberculosis suggests that there is an abundance of PG-hydrolyzing enzymes, it is not known whether these enzymes are indeed functionally redundant. We sought to investigate the physiological role of the PG amidase Ami1 and the endopeptidases RipA and RipB in M. tuberculosis. We discovered that the PG-hydrolyzing activity of a single amidase, Ami1, contributes to normal persistence of M. tuberculosis in the host but does not appear to play an important role in cell division *in vitro* under regular growth conditions. Ami1 does, however, contribute to the residual cell division activity in cells lacking the dominant PG hydrolase, RipA. Deletion of the d,l-endopeptidase RipA significantly impaired cell division in culture, and its depletion attenuated M. tuberculosis replication within macrophages and led to substantial killing of M. tuberculosis in mice. Furthermore, we observed that depletion of RipA renders M. tuberculosis more susceptible to several cell wall-targeting drugs and to the frontline anti-TB drug rifampin. Deletion of RipB did not affect cell division, yet the enzyme is essential in the absence of RipA. Together, this work shows that although multiple PG hydrolases are encoded in the M. tuberculosis genome, these enzymes fulfill important distinct roles in different contexts. This work may provide new strategies for the development of PG-targeting drugs against M. tuberculosis.

## RESULTS

### Ami1 is dispensable for cell division *in vitro* but participates in the maintenance of cell wall integrity and antibiotic resistance.

Deletion of the Ami1 homologue in M. smegmatis (MSMEG_6281) resulted in multiseptated chains with a high frequency of branching indicative of a cell division defect (18). To assess the importance of Ami1 for cell division in M. tuberculosis, we generated an Ami1 deletion (Δ*ami1*) mutant (see Fig. S1A and B in the supplemental material). Using the fluorescent d-alanine analogue (FDAA) HCC-amino-d-alanine (HADA) (20–22) that incorporates into newly synthesized peptidoglycan (Fig. 1B), we observed that Ami1 deficiency affected neither cell length nor the frequency of septa-containing cells (Fig. S1C and D). Nevertheless, M. tuberculosis lacking Ami1 or expressing an amidase-inactive mutant (Ami1_E200A_) (17) accumulated ethidium bromide faster and to a larger extent than the wild type or the mutant strain complemented with the wild-type enzyme (Fig. 1C). The levels of Ami1_E200A_ were confirmed to be similar to that of Ami1 expressed in the wild-type and complemented strains (Fig. S1E), demonstrating that the active site mutation did not affect protein stability. In line with the observed increased cell wall permeability, the M. tuberculosis Δ*ami1* mutant showed increased susceptibility to the frontline TB drug rifampin (Fig. 1D) and slightly increased susceptibility to the PG-targeting antibiotic vancomycin (see Fig. S2A in the supplemental material). Together, these data suggest that while Ami1 is dispensable for cell division in M. tuberculosis, the absence of this hydrolase leads to cell wall alterations, resulting in increased permeability and increased antibiotic susceptibility.

10.1128/mBio.03315-19.1 FIG S1 Validation of the M. tuberculosis Δ*ami1* mutant. (A) Schematic diagram of the *ami1* locus in wild-type (WT) and Δ*ami1* mutant bacteria. Red bars represent the labeled oligonucleotide probe. Labeling and detection of probe DNA were carried out using the ECL Direct nucleic acid labeling and detection system (GE Healthcare). (B) Southern blot of SacI-digested genomic DNA, probed with *ami1* horseradish peroxidase (HRP)-labeled probe. (C) Quantification of cell length measurements of WT and Δ*ami1* mutant populations. WT *n *= 90; Δ*ami1* mutant *n *= 113. The solid line represents the median. The area between the dashed lines represents the interquartile range. Significance was determined by the Kruskal-Wallis test; ns, not significant. (D) The percentage of the population of cells with septa. WT *n *= 113; Δ*ami1* mutant *n *= 131. Significance was determined by the Mann-Whitney test; ns, not significant. (E) Immunoblotting to confirm the expression of the amidase-inactive variant of Ami1 (Ami1_E200A_). Whole-cell lysates were prepared from wild-type (WT), and Δ*ami1* (M), Δ*ami1*::*ami1*_WT_ (C_WT_), and Δ*ami1*::*ami1*_E200A_ (C_E200A_) variant mid-log-phase cultures. Total protein (40 μg) was run by SDS-PAGE, and Ami1 protein was detected using rabbit anti-Ami1 sera. DlaT was used as a loading control. Download FIG S1, TIF file, 0.7 MB. Copyright © 2020 Healy et al. 2020 Healy et al. This content is distributed under the terms of the Creative Commons Attribution 4.0 International license.

10.1128/mBio.03315-19.2 FIG S2 Phenotypic analysis of Δ*ami1*. (A) MIC profiles of wild-type Δ*ami1*, Δ*ami1*::*ami1*_WT_, and Δ*ami1*::*ami1*_E200A_ strains for several cell wall-targeting drugs. (B) Bacterial burden in the spleens of CB57BL/6 mice infected by aerosol inhalation with the following strains: wild-type H37Rv (WT) and Δ*ami1*, Δ*ami1*::*ami1*_WT_, and Δ*ami1*::*ami1*_E200A_ variant. (C) CFU burden of the wild type (WT) and the Δ*ami1* mutant in naive and IFN-γ-activated murine BMDMs. Macrophages were infected at a multiplicity of infection (MOI) of 0.1. (D) Survival in phosphate-buffered saline (PBS) nutrient starvation model. (E) Survival during nitric oxide (NO) exposure for 3 days using diethylenetriamine NONOate (DETA-NO), supplemented every 24 hrs. All data are representative of at least two independent experiments. Data in panel B are from 2 independent experiments, each with 4 mice per group and time point. Download FIG S2, TIF file, 0.5 MB. Copyright © 2020 Healy et al. 2020 Healy et al. This content is distributed under the terms of the Creative Commons Attribution 4.0 International license.

### Ami1 amidase activity is required for persistence of M. tuberculosis in mice.

The lack of any obvious cell division phenotype of M. tuberculosis *Δami1* suggests that other PG amidases or hydrolases are compensating for the loss of Ami1. The enzyme may, however, be important under specific conditions, such as during infection. To test this, we infected mice with the *Δami1* mutant, the complemented mutant (Δ*ami1*::*ami1*) and an Δ*ami1* mutant expressing the inactive amidase (Δ*ami1*::*ami1_E200A_*) (17). The Δ*ami1* mutant replicated similarly to wild-type M. tuberculosis during the acute phase of infection (up to 28 days postinfection), but a decline in CFU burden was observed during the chronic phase of infection (Fig. 1E). This persistence defect was fully complemented by expressing Ami1 in the Δ*ami1* mutant background (Δ*ami1*::*ami1*_WT_). However, expression of Ami1_E200A_ did not complement the persistence defect, indicating that the amidase activity of Ami1 is important for persistence (Fig. 1E). Deletion of Ami1 did not have a significant impact on dissemination of M. tuberculosis to the spleen (Fig. S2B). This was surprising, as a transposon library screen predicted that Ami1 is important for persistence of M. tuberculosis in the spleen of mice (23); perhaps the persistence defect in the spleen is only revealed during competition with Ami1 expressing M. tuberculosis. Thus, the amidase activity of Ami1 is dispensable for replication during the acute phase of mouse infection but contributes to the ability of M. tuberculosis to persist in the lungs during the chronic phase of infection. We did not observe any attenuation of the M. tuberculosis Δ*ami1* mutant during macrophage infection (Fig. S2C), and the mutant survived nutrient deprivation and nitric oxide (NO) exposure, two host-relevant stresses, similarly to wild-type M. tuberculosis (Fig. S2D and E).

### Genome-wide fitness screening predicts synthetic lethality between Ami1 and RipA.

**FIG 2:**
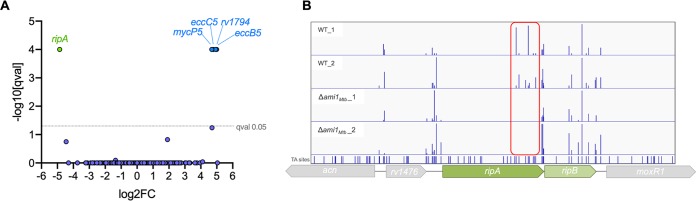
Transposon mutagenesis coupled with next-generation sequencing (Tn-seq) predicts synthetic lethality between *ami1* and *ripA*. (A) Identification of genetic interactors of *ami1*. For each gene, the ratio of normalized sequence reads per insertion site (Δ*ami1* pool/wild-type [WT] pool) is plotted on the *x* axis (log_2_ fold change). The *y* axis represents the significance of each of these changes in representation (*q* value). Data are from two biological replicate transposon mutant libraries per strain (B) Transposon insertion sites across the *ripAB* locus. The frequencies of TA dinucleotide sites with transposon insertions across the *ripAB* locus are represented by the height of the bars. The transposon insertions are shown for two independent replicate transposon mutant libraries generated in the wild type and the Δ*ami1* mutant. The red box highlights the insertions within the C-terminal hydrolase domain that are detected only in WT libraries.

To identify enzymes that compensate for the activity of Ami1 during growth in culture, we utilized transposon mutagenesis coupled with next-generation sequencing (Tn-seq). Transposon mutant libraries were generated in wild-type M. tuberculosis and the Δ*ami1* mutant, and the frequency of transposon mutants within the libraries was determined by sequencing the transposon-genome junctions to identify the insertion sites. We used the TRANSIT Tn-seq analysis tool (24) to identify mutants that were over- or underrepresented in the Δ*ami1* mutant libraries compared to the wild-type libraries (log_2_ fold change, greater than 2 or less than −0.5) with statistical significance after correction for multiple comparisons (*q* < 0.05). The enrichment of mutants in the Δ*ami1* mutant transposon library with disruption of genes encoding Esx5 type VII secretion system components (MycP5, EccB5, and EccC5) is in agreement with our finding that loss of Ami1 results in a leaky cell envelope, rendering the Esx5 system nonessential, as previously observed in Mycobacterium marinum (25). Surprisingly, only transposon insertions in a single gene, *ripA*, were identified as significantly underrepresented in the Δ*ami1* mutant libraries (Fig. 2A). RipA is a peptidoglycan d,l-endopeptidase that cleaves peptidoglycan in the peptide stem (Fig. 1A); it is thus not surprising that this enzyme can compensate for Ami1 activity. Interestingly, RipA is predicted to be essential for growth of M. tuberculosis (26). Our results show that transposon insertions occurred only within the C-terminal hydrolase-encoding domain of *ripA* (Fig. 2B). The complete lack of insertions in the N-terminal domain is likely because this domain regulates the hydrolase activity of RipA (C-terminal domain), which, if not controlled correctly, could severely damage the cell wall and lead to mortality (27). In order to confirm the predicted synthetic lethal relationship between RipA and Ami1, it was necessary to first determine whether RipA is essential in M. tuberculosis.

### RipA and RipB are not essential in M. tuberculosis.

**FIG 3:**
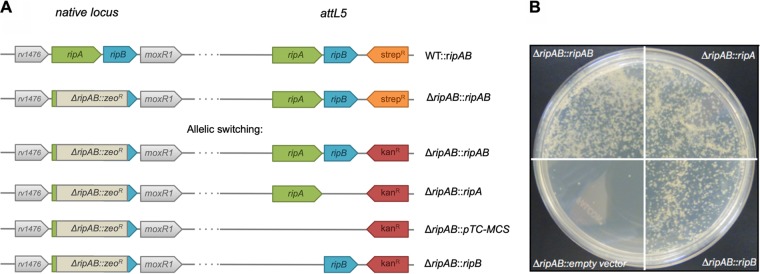
Allelic switching demonstrates that *ripA* and *ripB* are individually dispensable but that at least one of the two enzymes is required for viability. (A) Schematic diagram of the native *ripAB* locus and the *attL5* site for the various allelic switching strategies. (B) Photographs of agar plates of allelic switching transformations to replace the streptomycin resistance *ripAB* plasmid at the *attL5* locus with kanamycin resistance plasmids expressing both *ripA* and *ripB* (Δ*ripAB*::*ripAB*), *ripA* (Δ*ripAB*::*ripA*) or *ripB* (Δ*ripAB*::*ripB*) alone, or an empty plasmid (pTC-MCS).

A recent study in M. smegmatis demonstrated that *ripA* is not essential, provided that *ripB*, coding for another d,l-endopeptidase and located downstream of *ripA* within the same operon, is expressed (28). To examine whether *ripA* and *ripB* are essential in M. tuberculosis, we first generated a merodiploid strain by expressing the *ripA* and *ripB* genes from an integrated streptomycin resistance-conferring plasmid in the *attL5* locus (WT::*ripAB*) (Fig. 3A). Subsequently, the native *ripAB* locus was replaced by recombineering with a zeocin resistance cassette (Δ*ripAB*::*ripAB*). The recombination was confirmed by PCR targeting the junctions between the zeocin cassette and the adjacent genes at the native *ripAB* locus (see Fig. S3A and B in the supplemental material). Allelic switching experiments were carried out to replace the integrated plasmid at the *attL5* site containing the *ripAB* genes with plasmids expressing either *ripA* alone (Δ*ripAB*::*ripA*), *ripB* alone (Δ*ripAB*::*ripB*), or an empty plasmid (Δ*ripAB*::pTC-MCS) (Fig. 3A). Allelic switching with replacement plasmids expressing only *ripA* or *ripB* was successful (Fig. 3B). Several colonies were selected, and we confirmed by PCR that the *ripAB* plasmid was replaced with one that expressed a single *rip* endopeptidase gene (Fig. S3D). Attempts to replace the *ripAB* genes at the *attL5* site with an empty plasmid were unsuccessful (Fig. 3B; see also Fig. S3D, bottom). This confirmed that the PG endopeptidases RipA and RipB are individually dispensable in M. tuberculosis, but deletion of both *ripA* and *ripB* genes is either bactericidal or growth inhibitory for M. tuberculosis.

10.1128/mBio.03315-19.3 FIG S3 Confirmation of the replacement of the native *ripAB* locus with the Δ*ripAB*::*zeoR* cassette in an *ripAB* merodiploid background and subsequent allelic switching at the *attL5* locus. (A) Schematic diagram displaying the Δ*ripAB*::*zeoR* cassette at the native *ripAB* locus and the *ripAB* genes in the *attL5* integrated plasmid. Arrows indicate the primers used to confirm the Δ*ripAB*::*zeoR* cassette at the native *ripAB* locus. The line below indicates the DNA region used for recombination. (B). PCR results confirming two candidates using the primer pairs indicated below each gel image. (C) Kanamycin sensitivity check on cured Δ*ripAB*::*ripAB* candidates 3 and 4 (confirmed in panel B). Colonies picked after curing on 5% sucrose to ensure loss of the pNIT-sacB plasmid were checked for kanamycin sensitivity by spotting onto 7H10 agar plates with or without kanamycin. Candidate 3A was chosen to perform subsequent allelic switching experiments. (D) PCR to confirm the presence of the kanamycin resistance replacement plasmids at the *attL5* locus. The red asterisk indicates the confirmed candidate that was chosen for subsequent characterization. Download FIG S3, TIF file, 1.0 MB. Copyright © 2020 Healy et al. 2020 Healy et al. This content is distributed under the terms of the Creative Commons Attribution 4.0 International license.

### RipA is the major PG septal hydrolase required for cell division in M. tuberculosis.

**FIG 4:**
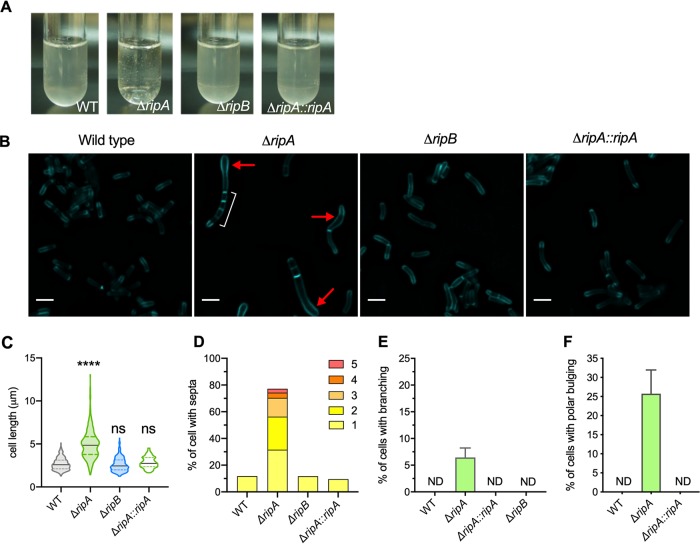
RipA is the major septal PG hydrolase required for cell division in M. tuberculosis. (A) Photographs of liquid cultures of the indicated strains. (B) Micrographs of cells labeled with HCC-amino-d-alanine (HADA) for 20 h. White bracket indicates multiple septa. Red arrow indicates polar bulging. Bar, 2 μm. (C) Violin plots showing the cell lengths of the WT (*n* = 312) and the Δ*ripA* (*n* = 268), Δ*ripB* (*n* = 210), and Δ*ripA*::*ripA* (*n =* 125) mutants. The black line represents the median; the area between the dashed lines is the interquartile range. Significance was determined by Kruskal-Wallis test; ****, *P* < 0.0001; ns, not significant. (D) Quantification of the proportion of cells with multiple septa in the WT (*n* = 361) and Δ*ripA* (*n* = 347), Δ*ripB* (*n* = 392), and Δ*ripA*::*ripA* mutants (*n* = 378). The bar height represents the percentage of cells that contain 1 or more septa. The colored segments within the bars represent the proportions of cells with different numbers of septa. (E) Quantification of the proportion of cells with branching. (F) Quantification of the proportion of cells with bulging in the polar region. ND, none detected. Data are representative of at least two independent experiments.

Upon culturing cells lacking RipA (Δ*ripAB*::*ripB*, which we will subsequently refer to as the Δ*ripA* mutant) in liquid medium, the cells were noticeably clumpy (Fig. 4A). We examined the cell division phenotype of cells lacking RipA by labeling the peptidoglycan with the fluorescent d-alanine analogue HADA. This revealed elongated multiseptated cells (Fig. 4B). Cells lacking RipA had an increased median cell length and a larger variation in cell length than the wild type and the complemented mutant (Δ*ripA*::*ripA*) (Fig. 4C). Approximately 10% of wild-type and Δ*ripA*::*ripA* mutant cells contained septa. In contrast, the majority of Δ*ripA* mutant cells (>70%) contained at least one septum, with half of that population containing more than one septum, indicating that M. tuberculosis without RipA elongates and forms septa but cannot efficiently separate into two daughter cells (Fig. 4D). Branching was also observed in a small fraction of cells lacking RipA, indicating a dysregulation of the PG elongation complex machinery that causes it to localize to the lateral cell wall instead of at the poles (Fig. 4E). However, further investigation is needed to confirm this hypothesis. Some Δ*ripA* cells contained bulges, most commonly at the poles, which might suggest that lack of RipA led to a weakened cell wall in the polar region, where the incorporation of new PG material occurs during cell elongation (Fig. 4F). In contrast, cells lacking RipB (Δ*ripB*) did not have any increase in cell length or in the frequency of cells with septa, indicating that RipB is dispensable for cell division in M. tuberculosis under regular *in vitro* conditions (Fig. 4).

### Loss of Ami1 further exaggerates the cell division defects of the M. tuberculosis Δ*ripA* mutant.

**FIG 5:**
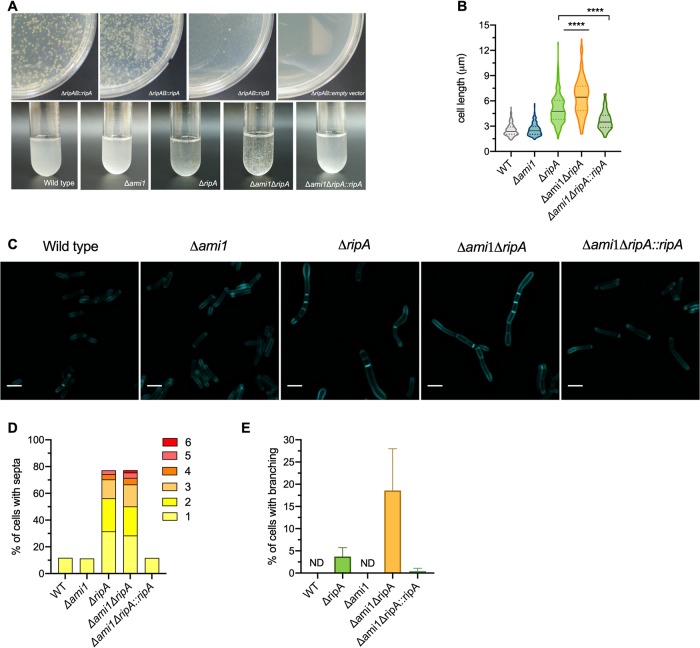
Deletion of *ami1* amplifies cell division defects in the Δ*ripA* mutant. (A) Agar plates of allelic switching transformations in the Δ*ami1* Δ*ripAB*::*ripAB* strain (top) and photographs of liquid cultures (bottom). (B) Violin plots of cell lengths for WT (*n* = 189) and Δ*ami1* (*n* = 187), Δ*ripA* (*n* = 196), Δ*ripA*::*ripA* (*n* = 143), Δ*ami1* Δ*ripA* (*n* = 214), and Δ*ami1* Δ*ripA*::*ripA* (*n *= 172) mutants. The black line represents the median; the area between the dashed lines is the interquartile range. Significance was determined by a Kruskal-Wallis test; ****, *P* < 0.0001. (C) Micrographs of cells labeled with HADA for 20 h. Bar, 2 μm. (D) Quantification of the proportion of cells with at least one septum. The bar height represents the percentage of cells that contain 1 or more septa. The colored segments within the bars represent the proportions of cells with different numbers of septa. WT (*n* = 195); Δ*ami1* (*n* = 380), Δ*ripA* (*n* = 206), Δ*ripA*::*ripA* (*n* = 314), Δ*ami1* Δ*ripA* (*n* = 225), and Δ*ami1* Δ*ripA*::*ripA* (*n* = 317) mutants. (E) Proportion of cells with branching. ND, none detected. Data in panels B to E are representative of two independent experiments.

After we established that RipA can be deleted from M. tuberculosis, we next examined the predicted synthetic lethality between RipA and Ami1 by attempting to delete *ripA* in the Δ*ami1* mutant background. We followed the same strategy as that described above, replacing the native *ripAB* with a zeocin resistance cassette in a *ripAB* merodiploid background of the Δ*ami1* mutant. Allelic switching with plasmids expressing one of either *rip* hydrolase gene was next attempted. Replacement of the *ripAB* genes with *ripB* alone resulted in very small, slow-growing colonies, suggesting that M. tuberculosis cells lacking both Ami1 and RipA are viable but severely compromised in growth (Fig. 5A, top). When the *ripAB* genes were replaced by *ripA* alone, the resulting colonies grew similarly to the positive control (where the integrated streptomycin resistance *ripAB*-expressing plasmid is replaced with a kanamycin resistance *ripAB*-expressing plasmid). Attempts to replace the integrated *ripAB* plasmid with an empty vector (pTC-MCS) were unsuccessful, suggesting that, as is the case in wild-type M. tuberculosis, at least one of the endopeptidases (RipA or RipB) is required for growth or viability of the Δ*ami1* mutant. Candidates resulting from each allelic replacement transformation were confirmed by PCR (see Fig. S4 in the supplemental material). Growth of the M. tuberculosis Δ*ami1* Δ*ripA* mutant in liquid medium was very slow, and the cells aggregated. The aggregates were noticeably bigger than those of the Δ*ripA* mutant (Fig. 5A, lower). M. tuberculosis Δ*ami1* Δ*ripA* mutant cells varied drastically in length, with a median length of 6.47 μm, which is significantly longer than that of cells lacking only RipA (4.75 μm) (Fig. 5B). Cells labeled with HADA showed elongated cells with multiple septa and a noticeable proportion of cells with branching (Fig. 5C and D). We observed more branching in Δ*ami1* Δ*ripA* mutant cells than in the Δ*ripA* mutant (Fig. 5E). Expression of RipA in Δ*ami1* Δ*ripA* (Δ*ami1* Δ*ripA*::*ripA*) rescued these phenotypes, further showing that RipA is required for efficient cell division. Deletion of *ripB* in the Δ*ami1* mutant background did not have any impact on cell division or length (see Fig. S5 in the supplemental material).

10.1128/mBio.03315-19.4 FIG S4 Confirmation of the replacement of the native *ripAB* locus with the Δ*ripAB*::*zeoR* cassette in the Δ*ami1 ripAB* merodiploid background and subsequent allelic switching at the *attL5* locus (A) Confirmation of the replacement of the native *ripAB* locus with a zeocin resistance cassette (upper panel). Red box indicates the confirmed candidate that was chosen for subsequent curing of the pNIT-sacB plasmid. Kanamycin sensitivity phenotype confirmation of the cured candidate (lower panel). (B) PCR to confirm the presence of the kanamycin resistance replacement plasmids at the *attL5* locus. The red asterisk indicates the confirmed Δ*ami1* Δ*ripB* mutant candidate that was chosen for subsequent characterization. Download FIG S4, TIF file, 0.6 MB. Copyright © 2020 Healy et al. 2020 Healy et al. This content is distributed under the terms of the Creative Commons Attribution 4.0 International license.

10.1128/mBio.03315-19.5 FIG S5 Cells lacking Ami1 and RipB do not have an increased cell length. Violin plots showing the cell lengths of the WT (*n* = 162) and the Δ*ami1* Δ*ripA* (*n* = 140) and Δ*ami1* Δ*ripB* (*n* = 89) mutants. The black line represents the median. The area between the dashed lines is the interquartile range. Significance was determined by the Kruskal-Wallis test; ****, *P* < 0.0001; ns, not significant. Data are representative of two independent experiments. Download FIG S5, TIF file, 0.1 MB. Copyright © 2020 Healy et al. 2020 Healy et al. This content is distributed under the terms of the Creative Commons Attribution 4.0 International license.

### RipA depletion increases the sensitivity of M. tuberculosis to multiple cell wall-targeting drugs and to the frontline TB drug rifampin.

**FIG 6:**
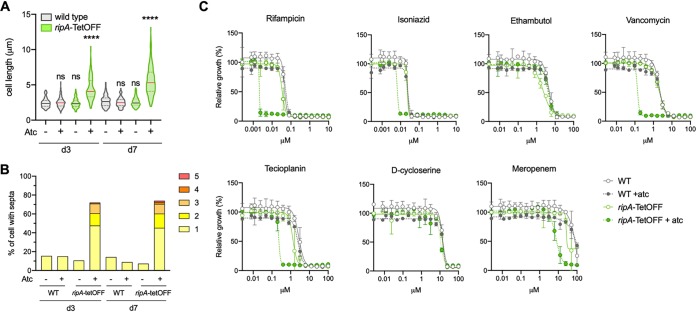
RipA depletion sensitizes M. tuberculosis to multiple cell wall-targeting drugs and to the frontline TB drug rifampin. (A) Violin plots of cell lengths upon *ripA* silencing with the addition of anhydrotetracycline (atc). Significance was determined by a Kruskal-Wallis test; ****, *P* < 0.0001; ns, not significant. (B) Quantification of the proportion of cells with at least one septum. The bar height represents the percentage of cells that contain 1 or more septa. The colored segments within the bars represent the proportions of cells with different numbers of septa. (C) MIC profiles of wild-type and *ripA*-tetOFF strains. Anhydrotetracycline (500 ng/ml) was added to cultures at the time of drug exposure. Growth was measured 14 days after addition of drug and atc. Data are representative of two independent experiments.

The poor growth and formation of aggregates of the M. tuberculosis Δ*ripA* mutant made it difficult to characterize the consequences of *ripA* deletion. We therefore generated a strain in which *ripA* could be transcriptionally silenced by the addition of anhydrotetracycline (atc) (*ripA-*TetOFF) (29). Silencing *ripA* slowed growth (see Fig. S6 in the supplemental material), and the bacteria formed elongated multiseptated chains similar to those of the Δ*ripA* mutant (Fig. 6A and B). We used *ripA-*TetOFF to determine whether the activities of frontline TB drugs and cell wall-targeting drugs that are active against mycobacteria are altered when RipA is depleted. Notably, the MICs of rifampin, isoniazid, teicoplanin, vancomycin, and meropenem were between 4-fold and 33-fold decreased when *ripA* was silenced (*ripA-*TetOFF + atc) (Fig. 6C). No shift in MIC was observed for ethambutol and d-cycloserine. Addition of atc did not alter the MICs against wild-type M. tuberculosis, showing that atc itself was not affecting the sensitivity of M. tuberculosis to the drugs tested.

10.1128/mBio.03315-19.6 FIG S6 Transcriptional silencing of *ripA* attenuates growth of M. tuberculosis. Growth curve of WT and *ripA*-TetOFF strains with or without 500 ng/ml anhydrotetracycline (atc). Data are representative of two independent experiments. Download FIG S6, TIF file, 0.1 MB. Copyright © 2020 Healy et al. 2020 Healy et al. This content is distributed under the terms of the Creative Commons Attribution 4.0 International license.

### RipA depletion leads to reduced replication in macrophages and to clearance of M. tuberculosis from infected mice.

**FIG 7:**
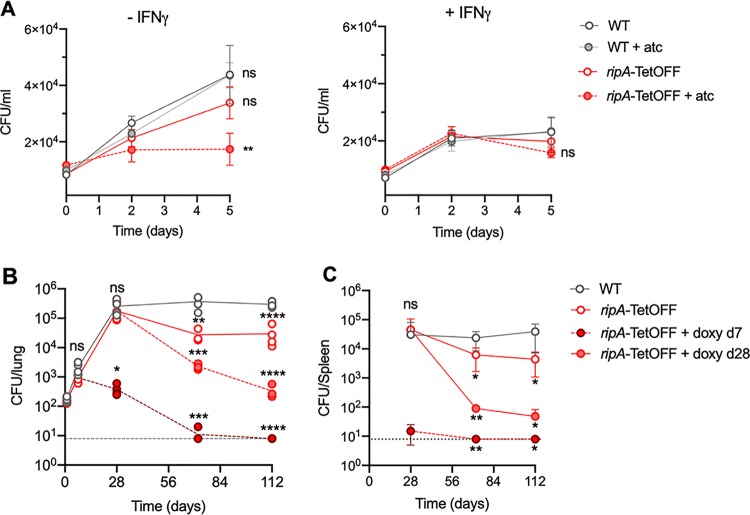
RipA depletion halts replication within macrophages and leads to clearance of M. tuberculosis from infected mice. (A) CFU quantification of the WT and the *ripA*-tetOFF (plus or minus atc) mutant during infection of naive and IFN-γ-activated murine BMDMs. Bacteria were incubated for 4 h at a multiplicity of infection (MOI) of 0.1 before washing to remove any nonphagocytosed bacteria. Macrophages were lysed in 0.01% Triton X-100, and the lysates were serially diluted and plated on 7H10 agar to determine the number of intracellular CFU. Anhydrotetracycline (atc) was added to wells at 4 h postinfection to silence *ripA* expression in intracellular M. tuberculosis. CFU burden in the lungs (B) and spleen (C) of mice infected by aerosol inhalation. The dashed lines indicate the limit of detection. At each time point, CFU of each strain was analyzed in groups of 4 mice. Doxycycline (doxy) was provided in chow to silence *ripA* expression in infected mice. The data are representative of two independent experiments. Significance was determined by one-way ANOVA and adjusted for multiple comparisons. ns, not significant; *, adj-*P* < 0.05; **, adj-*P* < 0.005; ***, adj-*P* < 0.0005; ****, adj-*P* < 0.0001.

Next, we determined the effects of RipA depletion on M. tuberculosis during infection of bone marrow-derived macrophages (BMDMs) *ex vivo* and during infection of mice. During macrophage infections, *ripA* silencing was initiated (by addition of atc to cell culture medium) after the bacteria had been phagocytosed by the macrophages (4 h postinfection [p.i.]). In resting BMDMs, *ripA* silencing led to growth inhibition, as the number of CFU remained unchanged during the 5-day infection period (Fig. 7A). Replication of *ripA*-TetOFF in the absence of atc was slightly lower than that of the wild type (plus or minus atc) but not significantly so. In interferon gamma (IFN-γ)-activated BMDMs, there was no significant difference in CFU between any of the strains, but a slight reduction in CFU of the RipA-depleted strain (*ripA-*TetOFF + atc) on day 5 p.i. was observed (Fig. 7A). RipA depletion thus halted replication of M. tuberculosis in macrophages but did not render the bacteria more prone to being killed upon macrophage activation. We next determined the effects of RipA depletion during infection in an animal model. We infected mice with wild-type and *ripA*-TetOFF strains by aerosol inhalation and induced depletion of RipA during the early acute phase of infection (day 7 p.i.) and also once the chronic phase of infection was established (day 28 p.i.) by feeding mice with chow containing doxycycline (doxy). Depleting RipA both at day 7 and day 28 lead to a reduction in CFU in the lungs and spleen (Fig. 7 B and C). Depleting RipA beginning on day 7 caused CFU to decline below the limit of detection by day 72. At day 112 p.i., the lungs from mice infected with the *ripA*-TetOFF mutant silenced at day 7 had no visible lesions, while those silenced at day 28 had very few lesions compared to the wild-type- and *ripA*-TetOFF mutant-infected lungs from mice that did not receive doxy (see Fig. S7 in the supplemental material). These data demonstrate that RipA is required for cell division *in vivo*, as silencing *ripA* expression halts M. tuberculosis replication within macrophages and prevents growth during mouse infection. Moreover, *ripA* silencing led to substantial killing of M. tuberculosis in chronically infected mice, suggesting that RipA is required for cell division during persistence and renders the bacteria susceptible to host-derived antibacterial stresses.

10.1128/mBio.03315-19.7 FIG S7 Gross pathology of mouse lungs infected with the *ripA*-TetOFF strain. The left upper lobes of infected mice at 112 days post infection were fixed in 4% formaldehyde. CFU data from these animals are shown in Fig. 7. Download FIG S7, TIF file, 0.9 MB. Copyright © 2020 Healy et al. 2020 Healy et al. This content is distributed under the terms of the Creative Commons Attribution 4.0 International license.

## DISCUSSION

The importance of PG for the survival and proliferation of bacteria is evident from the number of broad-spectrum antibiotics currently in use that target the enzymes that build and modify it. However, the multiplicity of enzymes synthesizing and/or modifying PG in M. tuberculosis and in other bacteria suggests redundancy between these enzymes and makes investigating the roles of individual enzymes difficult (13). Nevertheless, despite their similar enzymatic functions, these enzymes can be individually important for different processes of PG synthesis, degradation, and re-modeling across diverse environmental conditions. For example, two class A penicillin-binding proteins (PBPs), MrcB and MrcA, are specifically important for the growth of E. coli at acidic and alkaline pH, respectively (30). Salmonella enterica serovar Typhimurium expresses a PG synthase that is selectively active when Salmonella resides in acidic environments, including those inside macrophages (31). Our data reveal that Ami1 is specifically required in M. tuberculosis during chronic mouse infection yet completely dispensable for growth in culture. In contrast, M. smegmatis depends on Ami1 for normal cell division *in vitro* (18). M. smegmatis lacking RipA elongated and formed chains in acidic medium but was indistinguishable from wild-type M. smegmatis under regular culture conditions, indicating that RipA is dispensable for cell division in M. smegmatis unless the bacteria are exposed to acid stress (28, 32). However, in M. tuberculosis, we discovered that RipA is important for cell division during regular growth *in vitro* and during infection. There are notable differences in PG synthesis dynamics between M. smegmatis and M. tuberculosis (22), and our work reveals differences between the enzymes that functionally dominate septal separation during cell division in M. smegmatis and M. tuberculosis. Ami1 is the dominant septal PG hydrolase mediating cell division in M. smegmatis (18), while RipA fulfills this role in M. tuberculosis. Notwithstanding, M. tuberculosis and M. smegmatis both depend on either RipA or RipB for viability and replication. Their activities might not only be required for septal PG degradation that allows cell division but may also facilitate cell growth by cleaving PG at the polar region, allowing for the incorporation of new PG. It has been demonstrated that PG hydrolases are necessary for cell growth in other bacteria (33–35). The branching and polar bulging evident in Δ*ripA* and Δ*ami1* Δ*ripA* mutant cells suggest that RipA may also play a role in the incorporation or modification of new PG material during elongation that occurs at the poles in mycobacteria. Consistent with this hypothesis, RipA was shown to localize to the poles, as well as to the septa, in Mycobacterium bovis BCG (9). RipA is also known to interact with other cell wall enzymes. Its interaction with the lytic transglycosylase RpfB leads to a synergistic increase in their capacity to cleave PG (10). RipA also interacts with and is regulated by PonA1 (PBP1), a PG synthase that localizes to both the poles and septa and contributes to cell growth and virulence in mice (36, 37). The absence of RipA might thus lead to defects in cell growth and division not only by lack of its own enzymatic activity but also by perturbing its interacting partners, such as PonA1 and RpfB. This is supported by the branching observed in RipA-deficient cells, which suggests a disturbance in the localization of enzymes that normally congregate to the poles and elongate the sidewall.

The amidase activity of Ami1 contributes to the ability of M. tuberculosis to persist within the host, most likely by supporting cell division in a bacterial subpopulation, as the stable bacterial burden during the chronic phase of infection has been demonstrated to be a result of balanced replication and death (38). Previous work in our lab identified an integral membrane protein involved in cell division, PerM, that is also specifically required for persistence in mice (39, 40). RipA is essential for replication in the acute phase of infection and also during persistence in the chronic phase. Together, these findings indicate that M. tuberculosis depends on replication and/or PG turnover to survive during chronic infection. We speculate that during persistence *in vivo*, a specific cell division or cell envelope modification complex forms that, in addition to the dominant PG hydrolase RipA, also includes Ami1, PerM, and other enzymes that strengthen PG against immune-driven host stresses.

Our work demonstrates that RipA represents an attractive drug target, as its inhibition results in clearance of M. tuberculosis during infection and renders M. tuberculosis significantly more vulnerable to drugs currently used to treat TB, as well as to PG-targeting glycopeptides. Furthermore, its localization in the periplasm facilitates access to inhibiting compounds. Despite the multitude of d,l-endopeptidases and amidases in M. tuberculosis, we found that RipA and Ami1 are individually important. We hope that a greater understanding of the specific roles of individual enzymes that synthesize and modify PG will motivate the development of novel cell wall-targeting drugs against M. tuberculosis.

## MATERIALS AND METHODS

### Bacterial cultures and manipulation.

Mycobacteria were grown in liquid Middlebrook 7H9 medium supplemented with 0.2% glycerol, 0.05% tyloxapol, and ADN (0.5% bovine serum albumin, 0.2% dextrose, 0.085% NaCl) and on solid agar plates with Middlebrook 7H10 agar supplemented with 0.2% glycerol and Middlebrook oleic acid-albumin-dextrose-catalase (OADC) enrichment (Becton, Dickinson). The following antibiotics were added for selection of genetically modified strains at the following concentrations: hygromycin (50 μg/ml), kanamycin (25 μg/ml), streptomycin (25 μg/ml), and zeocin (25 μg/ml); anhydrotetracycline (500 ng/ml) was added to induce gene silencing. The strains used in this study are listed in Table S1 in the supplemental material.

10.1128/mBio.03315-19.8 TABLE S1 List of strains used in this study. Download Table S1, PDF file, 0.03 MB. Copyright © 2020 Healy et al. 2020 Healy et al. This content is distributed under the terms of the Creative Commons Attribution 4.0 International license.

### Mutant generation and complementation.

Knockout mutants were generated in M. tuberculosis H37Rv by recombineering as previously described (41, 42). All plasmids were generated using Gateway cloning technology (Life Technologies). The knockout cassettes for *ami1* (*rv3717*) and *ripAB* (*rv1477* and *rv1478*) consisted of 500 bp upstream and 500 bp downstream of the genes to be replaced. *ami1* was replaced, apart from the first and last 20 bp of the gene, by a hygromycin resistance cassette (see Fig. S1 in the supplemental material). The *ripA* and *ripB* genes were replaced with a zeocin resistance cassette. The knockout cassettes were cut from their vector backbones by restriction digest and transformed into the recombineering strain (H37Rv::pNIT-RecET) (42, 43). Recombinants were selected on 7H10 agar plates containing the appropriate antibiotics. Recombinant candidates were confirmed by PCR and then cured of the pNIT-RecET plasmid by counterselection on 5% sucrose 7H10 agar plates and confirmed by kanamycin sensitivity (see Fig. S3C and S4A in the supplemental material). Complementation of Δ*ami1* was achieved by expressing *ami1* under the control of its native promoter (200 bp upstream of *ami1*). Complementation with *ripA* and *ripB* was achieved by expressing either both genes or a single *rip* gene under the control of a constitutive promoter (Pmyc1-tetO [44]). The plasmids used in this study are listed in Table S2 in the supplemental material.

10.1128/mBio.03315-19.9 TABLE S2 Plasmids used in this study. Download Table S2, PDF file, 0.02 MB. Copyright © 2020 Healy et al. 2020 Healy et al. This content is distributed under the terms of the Creative Commons Attribution 4.0 International license.

### Generation of Ami1 antiserum and immunoblotting.

An N-terminal hexahistidine-tagged *rv3717* (*ami1*) gene fusion was generated by cloning the *rv3717* gene (without the secretion signal peptide) into the pET-300/NT-DEST plasmid. Expression of the His-tagged Ami1 protein in the BL21 (DE3) strain of E. coli was induced at 25°C for 6 h before purification using a His Trap high-performance (HP) column. The purified protein was checked by SDS-PAGE and then concentrated and dialyzed against phosphate-buffered saline (PBS). Rabbit polyclonal antiserum against recombinant Ami1 was generated by Thermo Fisher Scientific.

### Transposon mutant library sequencing.

Transposon mutant libraries were constructed in wild-type H37Rv and the Δ*ami1* mutant by *himar1* mutagenesis as described previously (43). Genomic DNA was extracted from the transposon libraries, and the library mutant composition was determined by sequencing amplicons of the transposon-genome junctions as described previously (26, 45). Mapping and quantification of transposon insertions was done as described previously (26, 43). Genes affecting the fitness/viability in the Δ*ami1* mutant background were identified using the resampling test module in the TRANSIT analysis platform (24). *P* values were defined as the proportion of values within 10^6^ permutations that had a value more extreme than the observed experimental result, and these *P* values were adjusted for multiple comparisons (*q* value) using the Benjamini-Hochberg procedure. Tn-seq fold changes (TnSeq-FC) were computed as log_2_-transformed ratios of the normalized read counts between the wild type and Δ*ami1* mutant libraries. We defined genes having a *q* value of <0.05 according to the permutation test to be significant determinants of fitness in the absence of *ami1*.

### Peptidoglycan labeling with fluorescent d-alanine analogues.

Fluorescent d-alanine analog (FDAA) HADA (HCC-amino-d-alanine) was synthesized by the Memorial Sloan Kettering Cancer Center (MSKCC) Organic Synthesis Core Facility following the procedure described by Kuru and colleagues (21). For incorporation of fluorescent d-alanine analogues into the peptidoglycan of M. tuberculosis, cultures with an optical density at 580 nm (OD_580_) of 0.1 to 0.3 were incubated in 7H9 medium containing 1 mM HADA at 37°C for the indicated times. Cells were washed three times in PBS-0.05% tyloxapol and fixed with 4% formaldehyde.

### High-resolution microscopy.

Microscopy imaging was performed using equipment and methods described previously (22). Fixed bacterial suspensions were spread onto soft agar pads (7H9 medium plus 1% agarose) and visualized with the appropriate filter set. Training and advice were provided at the Bio-Imaging Resource Center at Rockefeller University. Images were analyzed using ImageJ software.

### Mouse infection.

The animal experiments were performed in accordance with National Institutes of Health guidelines for housing and care of laboratory animals and according to institutional regulations after protocol review and approval by the Institutional Animal Care and Use Committee of Weill Cornell Medicine (protocol number 0601441A). Female 7- to 8-week-old C57BL/6 mice (Jackson Laboratory) were infected with ∼100 CFU using an inhalation exposure system (Glas-Col). CFU burden of lungs and spleens at each time point was determined by plating dilutions of organ homogenates on 7H10 agar. Four mice were euthanized at each time point for each group. For *ripA* silencing *in vivo*, mice were provided with chow containing doxycycline (2,000 ppm; Research Diets).

### Preparation and infection of murine bone marrow-derived macrophages.

Femurs and tibias of female C57BL/6 mice were extracted, and bone marrow cells were aseptically flushed using PBS. Cells were resuspended in Dulbecco’s modified eagle medium (DMEM) supplemented with 10% (vol/vol) fetal bovine serum (FBS), 10 mM HEPES, and 20% (vol/vol) L929 culture filtrate (LCM) and incubated for 6 days to allow differentiation into macrophages. Cells were harvested and seeded at 6 × 10^4^ cells per well in 96-well plates in 10% LCM complete DMEM overnight before infection. Macrophages were activated with IFN-γ (20 ng/ml) overnight before infection and throughout the infection. Mycobacteria were washed in PBS + 0.05% tyloxapol, and a single cell suspension was generated by low-speed centrifugation to pellet clumped cells. The bacteria were diluted into 10% LCM and added to macrophages at a multiplicity of infection (MOI) of 0.1. After 4 h, extracellular bacteria were removed by washing the macrophages three times with warm PBS. The number of intracellular bacteria was determined by lysing macrophages with 0.01% Triton X-100 and culturing serial dilutions of macrophage lysates on 7H10 agar plates.

### Determination of the MIC.

Cultures were grown to the mid-log phase and washed once in 7H9 medium, and a single cell suspension was prepared by low-speed centrifugation to pellet clumps. Single cell suspensions were used to inoculate 384-well black plates with clear flat bottoms that contained a range of drug concentrations. Drugs were dispensed using an HP D300e Digital Dispenser (Hewlett Packard). The drug dispensing was randomized using the HP Digital Dispenser software (version 3.2.2), and the dimethyl sulfoxide (DMSO) concentration in each well was normalized to 1%. After incubation at 37°C (5% CO_2_) for 12 days, the optical density (OD_580_) in each well was read using a SoftMax M2 plate reader. The data were derandomized using HP Digital Dispenser Data Merge software. The percentage of growth relative to that of the no-drug controls was calculated for each drug.
