# Supplementary figures and images for: Peptidoglycan Hydrolases RipA and Ami1 Are Critical for Replication and Persistence of Mycobacterium tuberculosis in the Host
Source: mBio. 2020 Mar 3;11(2):e03315-19. doi: 10.1128/mBio.03315-19 (PMC7064781; doi:10.1128/mBio.03315-19)

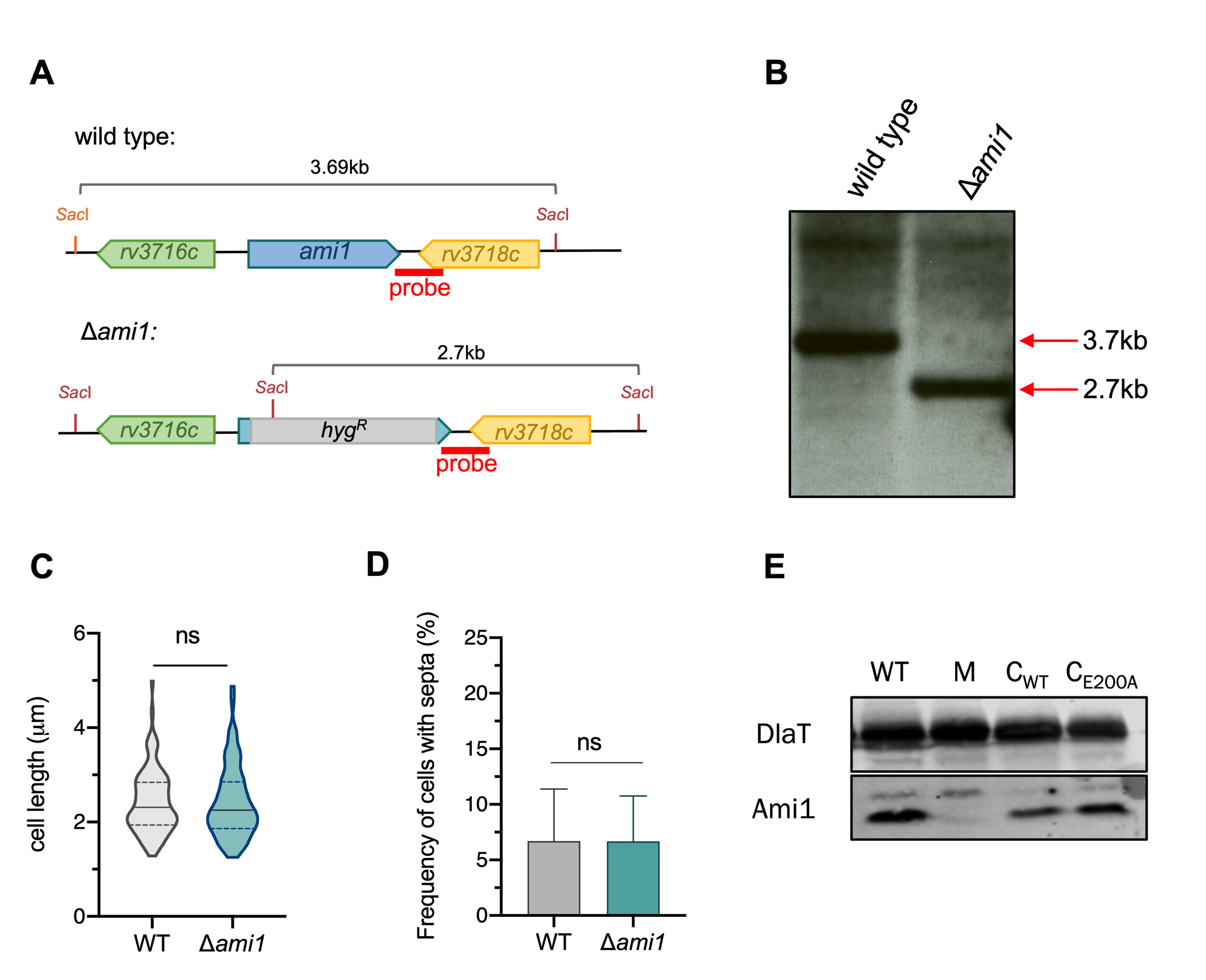

Supplement: FIG S1 [file mBio.03315-19-sf001.tif]

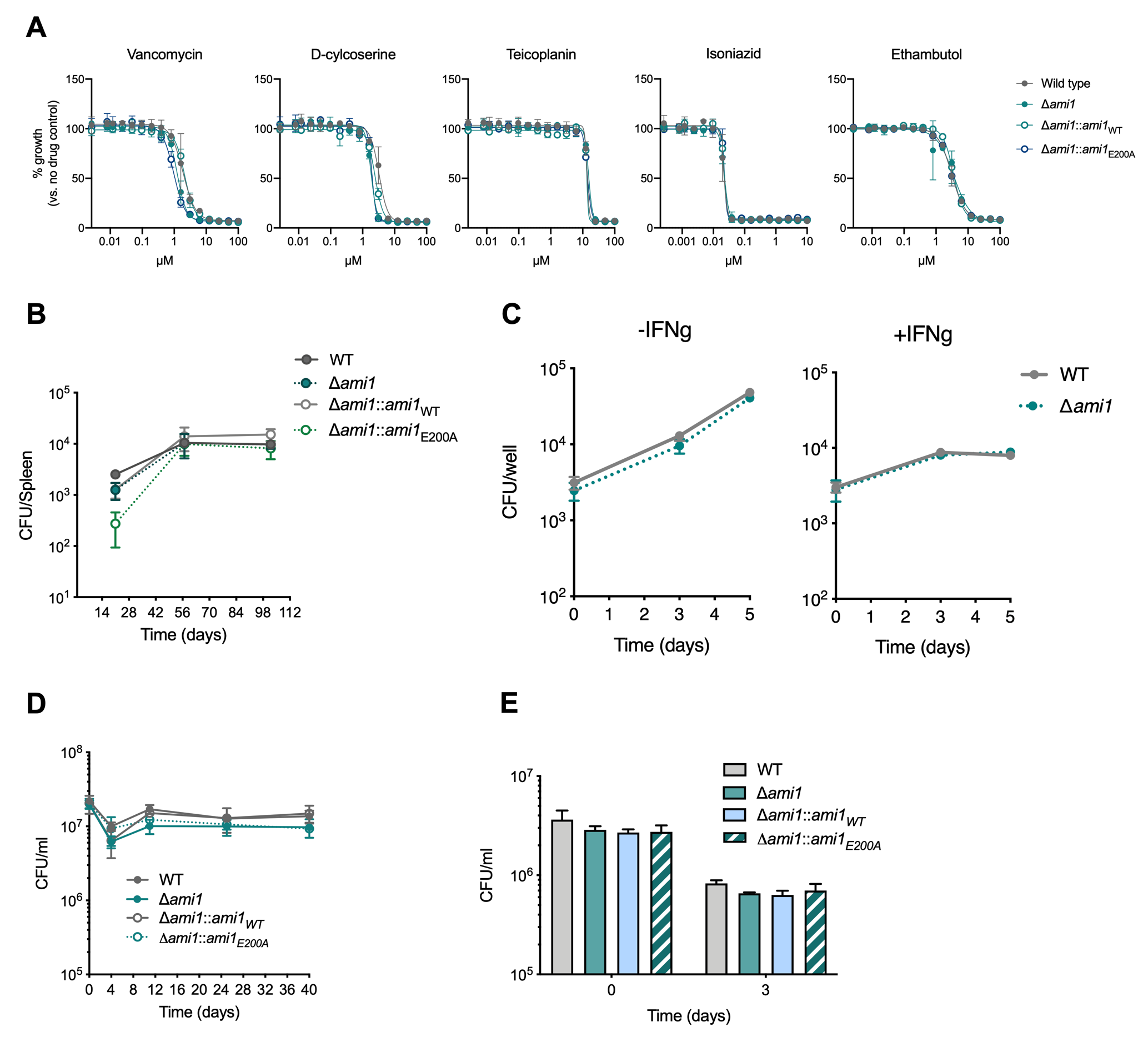

Supplement: FIG S2 [file mBio.03315-19-sf002.tif]

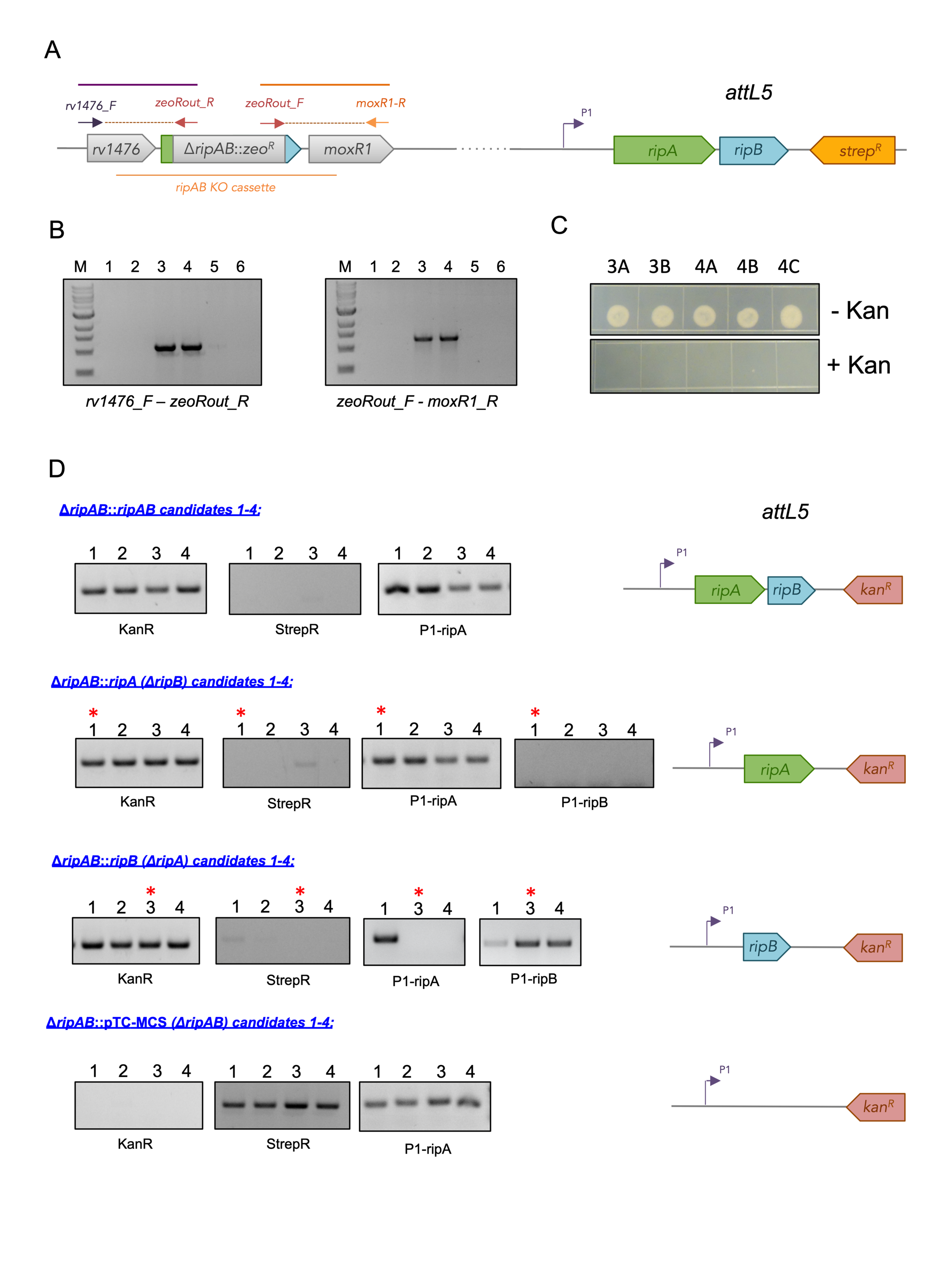

Supplement: FIG S3 [file mBio.03315-19-sf003.tif]

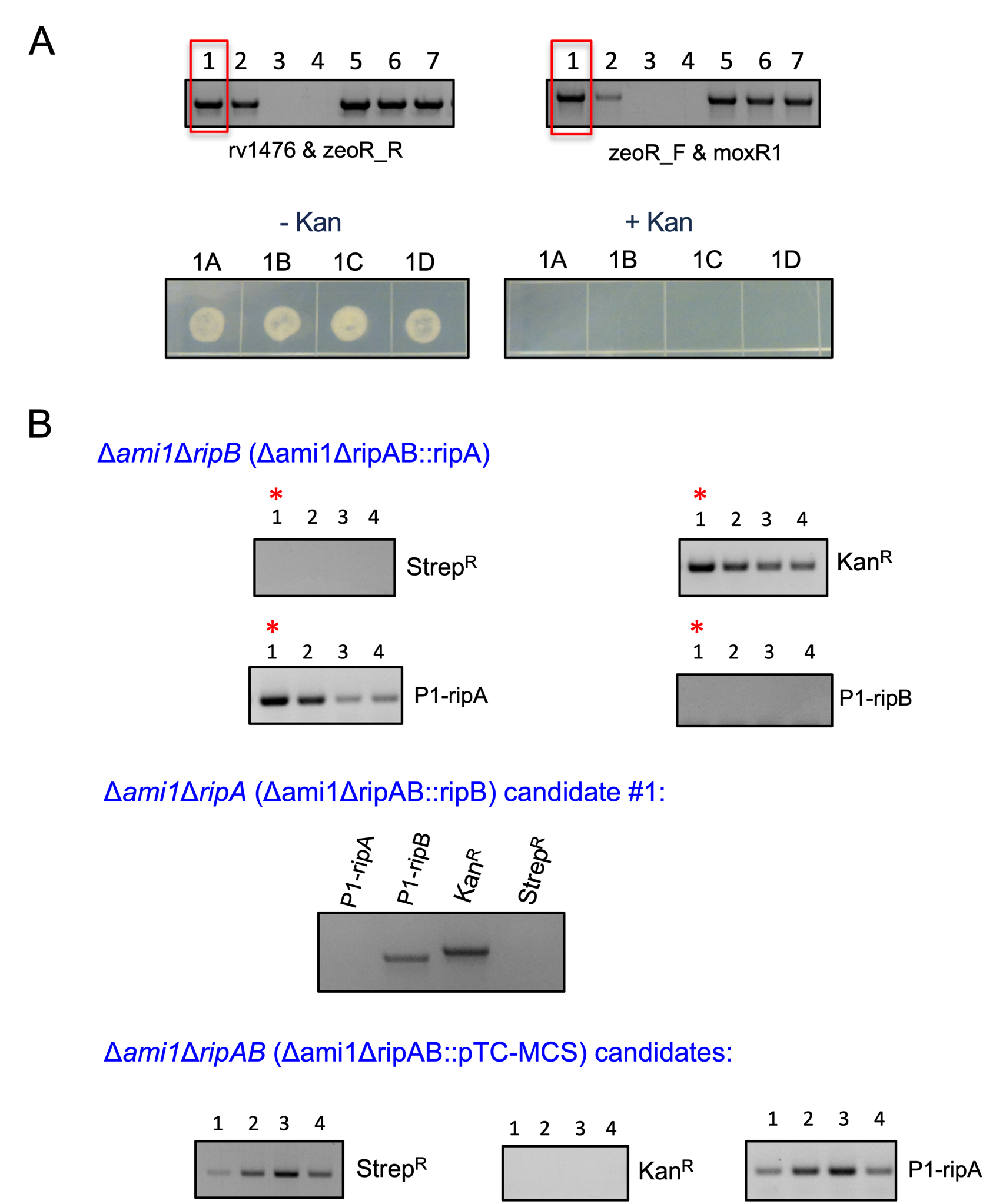

Supplement: FIG S4 [file mBio.03315-19-sf004.tif]

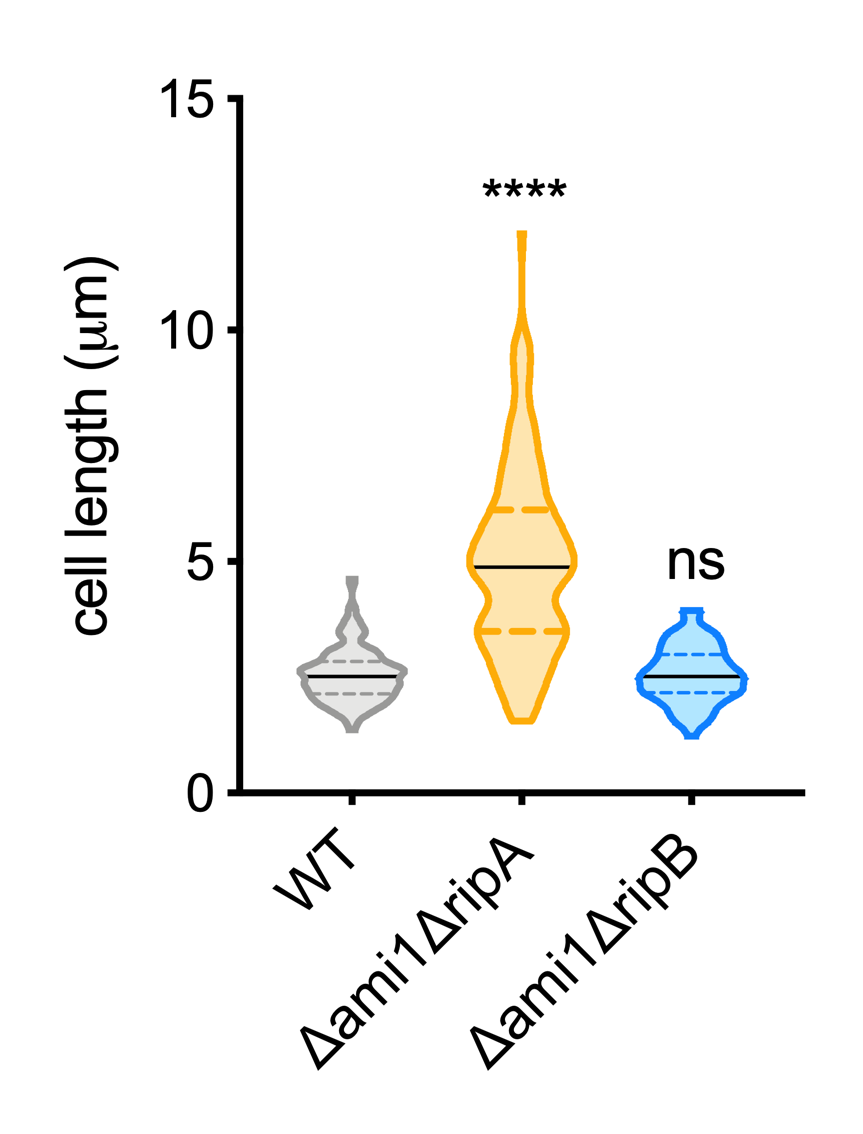

Supplement: FIG S5 [file mBio.03315-19-sf005.tif]

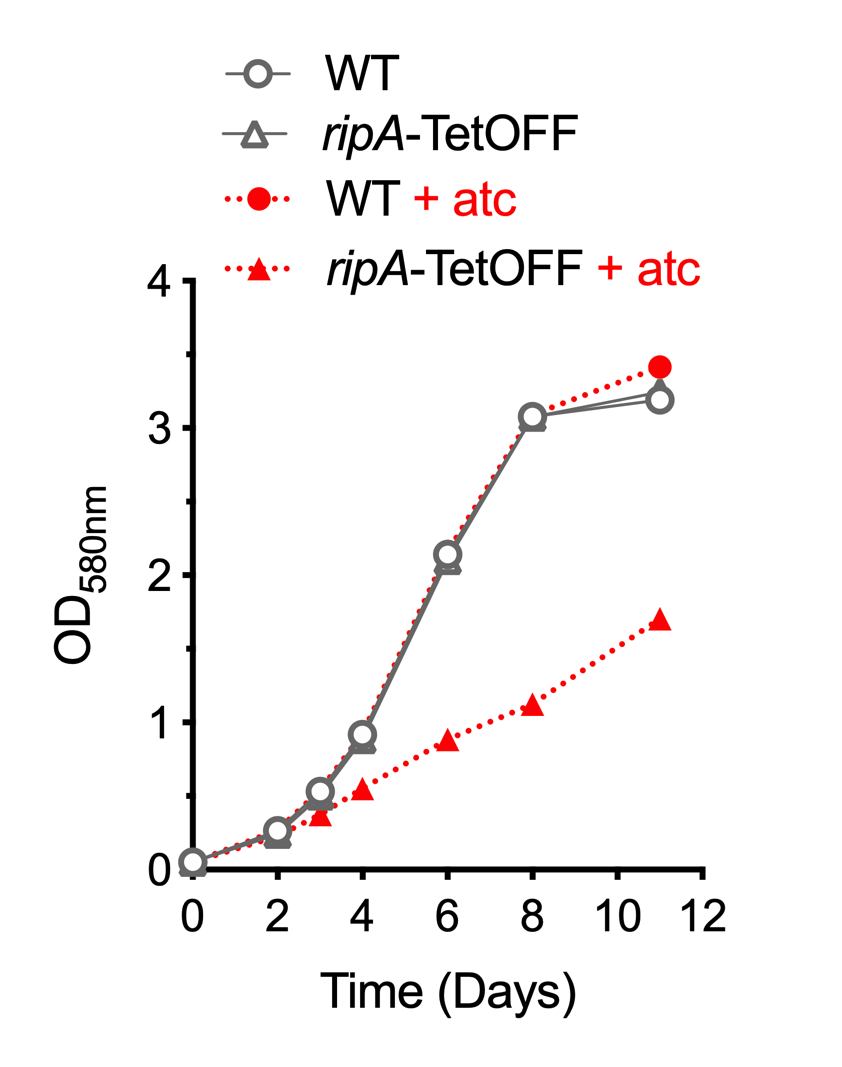

Supplement: FIG S6 [file mBio.03315-19-sf006.tif]

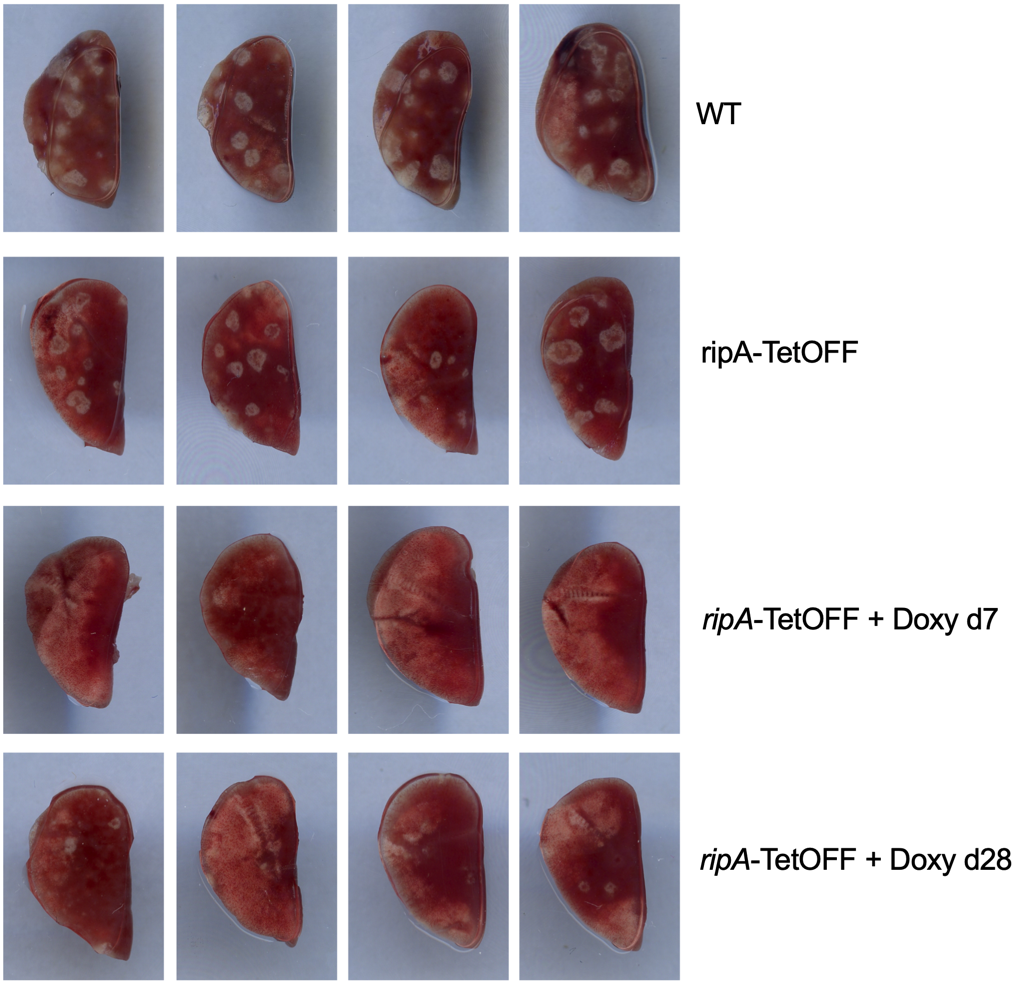

Supplement: FIG S7 [file mBio.03315-19-sf007.tif]
