# Supplementary material for: Peptidoglycan Hydrolases RipA and Ami1 Are Critical for Replication and Persistence of Mycobacterium tuberculosis in the Host
Source: mBio. 2020 Mar 3;11(2):e03315-19. doi: 10.1128/mBio.03315-19 (PMC7064781; doi:10.1128/mBio.03315-19)
Supplement: TABLE S1 [file mBio.03315-19-st001.pdf]

Table S1. List of strains used in this study

	Genotype	Source
**H37Rv**	H37Rv, wild type	Gift from C. Sassetti
Δami1	Δ*ami1*::*hygR*	This study
**Δ*ami1*::*ami1*^{WT}**	Δ*ami1*::*hygR*::pGMCK-*Pnat-ami1*	This study
**Δ*ami1*::*ami1*^{E200A}**	Δ*ami1*::*hygR*::pGMCK-*Pnat-ami1*$_{E200A}$	This study
**WT::*ripAB***	pGMCK-*P1-ripA-ripB*	This study
**Δ*ripAB*::*ripAB***	Δ*ripAB*::*zeoR*::pGMCS-*P1-ripA-ripB*	This study
**Δ*ripA***	Δ*ripAB*::*zeoR*::pGMCK-*P1-ripB*	This study
**Δ*ripB***	Δ*ripAB*::*zeoR*::pGMCK-*P1-ripA*	This study
**Δ*ripA::ripA***	Δ*ripAB*::*zeoR*::pGMCK-*P1-ripB*; Giles: pGMCgS-*P1-ripA*	This study
**Δami1::*ripAB***	Δ*ami1*::*hygR* pGMCK-*P1-ripA-ripB*	This study
**Δ*ami1*Δ*ripA***	Δ*ami1*::*hygR* Δ*ripAB*::*zeoR*::pGMCK-*P1-ripB*	This study
**Δ*ami1*Δ*ripA*::*ripA***	Δ*ami1*::*hygR* Δ*ripAB*::*zeoR*::pGMCK-*P1-ripB* pGMCgS-*P1-ripA*	This study
**Δ*ami1*Δ*ripB***	Δ*ami1*::*hygR* Δ*ripAB*::*zeoR*::pGMCK-*P1-ripA*	This study
***ripA*-TetOFF**	*ΔripAB*::*zeoR*::pGMCK-*P1-ripB*, Giles: pGMCgS-*T38S38-P750-ripA-HA*	This study
