# Supplementary material for: Peptidoglycan Hydrolases RipA and Ami1 Are Critical for Replication and Persistence of Mycobacterium tuberculosis in the Host
Source: mBio. 2020 Mar 3;11(2):e03315-19. doi: 10.1128/mBio.03315-19 (PMC7064781; doi:10.1128/mBio.03315-19)
Supplement: TABLE S2 [file mBio.03315-19-st002.pdf]

Table S2. Plasmids used in this study

Name	Description	Integration site	Resistance
**pGMCK-*Pnat-ami1$_{WT}$***	Expression of *ami1* from native promoter	L5	Kan
**pGMCK-*Pnat-ami1$_{E200A}$***	Expression of *ami1*$_{E200A}$ (inactive amidase) from native promoter	L5	Kan
**pGMCS-*P1-ripA-ripB***	Expression of *ripA* and *ripB* from Pmyc1-tetO constitutive promoter	L5	Strep
**pGMCK-*P1-ripA-ripB***	Expression of *ripA* and *ripB* from Pmyc1-tetO constitutive promoter	L5	Kan
**pGMCK-*P1-ripA***	Expression of *ripA* from Pmyc1-tetO constitutive promoter	L5	Kan
**pGMCK-*P1-ripB***	Expression of *ripB* from Pmyc1-tetO constitutive promoter	L5	Kan
**pTC-MCS**	empty vector	L5	Kan
**pGMCgS-*P1-ripA***	Expression of *ripA* from Pmyc1-tetO constitutive promoter	Giles	Strep
**pGMCgS-*T38S38-P750-ripA-HA***	Transcriptional repression of *ripA* with atc (TetOFF)	Giles	Strep
